# Genetic and Epigenetic Alterations Underlie Oligodendroglia Susceptibility and White Matter Etiology in Psychiatric Disorders

**DOI:** 10.3389/fgene.2018.00565

**Published:** 2018-11-22

**Authors:** Xianjun Chen, Huifeng Duan, Lan Xiao, Jingli Gan

**Affiliations:** ^1^Department of Psychiatry, Mental Diseases Prevention and Treatment Institute of PLA, PLA 91st Central Hospital, Jiaozuo, China; ^2^Department of Histology and Embryology, Chongqing Key Laboratory of Neurobiology, Army Medical University (Third Military Medical University), Chongqing, China

**Keywords:** psychiatric disorders, schizophrenia, oligodendroglia, myelin, white matter, genetic risk loci, epigenetic dysregulation

## Abstract

Numerous genetic risk loci are found to associate with major neuropsychiatric disorders represented by schizophrenia. The pathogenic roles of genetic risk loci in psychiatric diseases are further complicated by the association with cell lineage- and/or developmental stage-specific epigenetic alterations. Besides aberrant assembly and malfunction of neuronal circuitry, an increasing volume of discoveries clearly demonstrate impairment of oligodendroglia and disruption of white matter integrity in psychiatric diseases. Nonetheless, whether and how genetic risk factors and epigenetic dysregulations for neuronal susceptibility may affect oligodendroglia is largely unknown. In this mini-review, we will discuss emerging evidence regarding the functional interplay between genetic risk loci and epigenetic factors, which may underlie compromised oligodendroglia and myelin development in neuropsychiatric disorders. Transcriptional and epigenetic factors are the major aspects affected in oligodendroglia. Moreover, multiple disease susceptibility genes are connected by epigenetically modulated transcriptional and post-transcriptional mechanisms. Oligodendroglia specific complex molecular orchestra may explain how distinct risk factors lead to the common clinical expression of white matter pathology of neuropsychiatric disorders.

## Introduction

The highly complex etiology of neuropsychiatric disorders, which is influenced by both genetic predisposition and environmental factors, has been a major challenge in understanding these devastating diseases, like schizophrenia. In recent years, genomic studies have uncovered the complex genetic architecture of psychiatric disorders including thousands of genetic loci (Sullivan et al., 2012). Multiple genome-wide association studies (GWAS) have further produced remarkable findings in different populations. Besides, rare mutations, which are sufficiently deleterious with a low frequency, also play important roles in the pathogenesis (Abecasis et al., 2012). These advances in genetics have updated our understanding of psychiatric disorders. However, since some common symptoms of major psychiatric diseases have already been well-defined, how numerous genetic alterations affect function of multiple distinct genes and thereafter lead to similar clinical phenotypes is a puzzle.

Apart from the genetic component, environmental risk factors, including biological and psychosocial ones, are also involved in mental diseases onset (Owen et al., 2016). Environmental insult induces stable changes in gene expression, which are governed by epigenetic modifications (Nestler et al., 2016). Epigenetic modifications, including DNA methylation, histone modifications, and non-coding RNAs, played a functional control over the genetic information by regulating chromatin accessibility and gene transcription (Shorter and Miller, 2015). Chromatin modification analysis (assay for transposase accessible chromatin followed by sequencing, ATAC-seq) and transcriptome evidence suggest a possible molecular framework of how genetic alterations and epigenetic factors interact with each other (Guo et al., 2017; Fan et al., 2018).

Decades of extensive investigations revealed aberrant neuronal circuit assembly and synaptic malfunction of neuropsychiatric diseases, which forms the mechanisms for most current antipsychotics and new therapeutic development (Forrest et al., 2018). Recent studies of genetic alterations strongly support a neuronal susceptibility of psychiatry diseases. A large-scale GWAS has also implicated common variation in genes encoding the glutamate receptors, dopamine receptors, and members of voltage-gated calcium channel family of proteins (2014). Studies for rare mutations implicate that genes encoding a variety of synaptic proteins and the above-mentioned voltage-gated calcium channel related proteins are involved in the pathogenesis (Hall et al., 2015). Besides, epigenetic alterations underlying aberrant gene regulation in neurons have also been implicated in psychiatric disorders (Tsankova et al., 2007; Iwamoto and Kato, 2009; Guidotti et al., 2016).

Moreover, in recent years, neuropathological, neuroimaging, and genetic studies clearly revealed developmental defects in oligodendroglia/myelin formation and disrupted white matter integrity in major psychiatric disorders, including schizophrenia, depression, and bipolar disorders (Fields, 2008; Martins-de-Souza, 2010; Edgar and Sibille, 2012). Postmortem and brain imaging evidence showed volume reduction and ultrastructural changes of white matter in the prefrontal cortex of schizophrenic patients (Sanfilipo et al., 2000; Staal et al., 2000; Van Haren et al., 2004; Thong et al., 2014). Moreover, several postmortem studies revealed a loss of oligodendroglia in multiple brain regions (Hof et al., 2003; Stark et al., 2004; Byne et al., 2008), as well as some apoptotic and necrotic signs in oligodendroglia (Uranova et al., 2001). Another study further identified a decrease in the total number of oligodendroglia lineage cells, but not the number of progenitor cells, implying impaired differentiation of oligodendroglia (Mauney et al., 2015). In terms of schizophrenia, even before disease onset, the impaired myelin integrity occurs in frontal areas and advances in further stages to more brain regions (Friedman et al., 2008; Yao et al., 2013; Holleran et al., 2014; Liu et al., 2014), suggesting that oligodendroglia and myelin deficits are involved in the early pathogenesis. Besides, some neurological disorders characterized by white matter abnormalities, such as leukodystrophies and multiple sclerosis, showed some psychosis symptoms (Walterfang et al., 2005; Mckay et al., 2018). Rodent models with impaired oligodendroglia development and myelin deficits exhibit various phenotypes reminiscent of psychiatric disorders (Chen et al., 2015; Poggi et al., 2016). Importantly, some clinical studies found that the abnormal frontal myelin integrity was correlated to cognitive symptoms in first episode patients with schizophrenia (Perez-Iglesias et al., 2010; Kuswanto et al., 2012). These results strongly suggest that impaired oligodendroglia development and myelination could be related to the etiology of psychiatric disorders rather than simply being accompanied pathological abnormalities.

However, several questions need further discussion. How do genetic alterations and epigenetic dysregulations influence oligodendroglia besides neurons? Are there any cell-type-specific or developmental-stage-specific mechanisms in oligodendroglia? Whether and how do multiple risk factors coordinate to play roles in oligodendroglia? In this mini-review, we will try to address these questions based on emerging concepts of genetic and epigenetic findings in oligodendroglia during the pathogenesis of psychiatric disorders.

## Genetic Alterations Associated With Schizophrenia are Predicted to Affect Coding Genes Expressed in Both Neurons and Oligodendroglia

**FIGURE 1:**
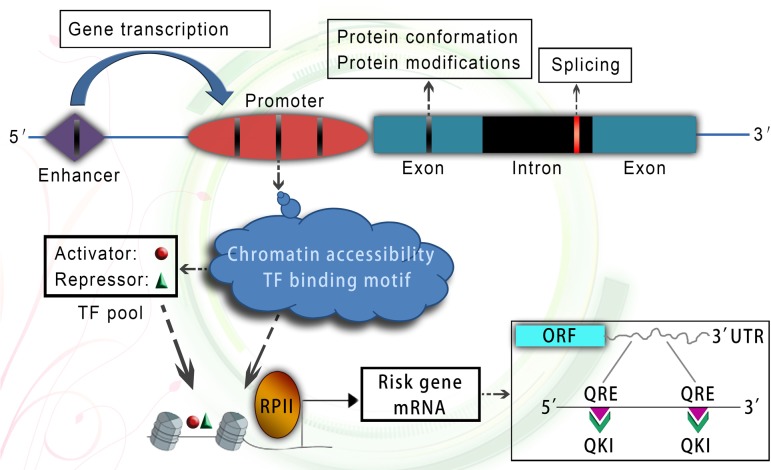
A working model of genetic alterations and epigenetic molecular network. Genetic variants occurring in coding region could change protein conformation and post-translational modifications. Genetic variants occurring in splicing related site located in intron could cause aberrant splicing. Genetic variants occurring in enhancer and promoter region could affect gene transcription through changing chromatin accessibility and TF binding motif. Epigenetically dysregulated chromatin accessibility of risk gene promoter and multiple TFs could coordinate to control risk gene transcription. In addition, the glia-specific RNA-binding protein quaking can stabilize risk gene mRNA at post-transcriptional level in oligodendroglia. TF, transcription factor; ORF, open reading frame; UTR, untranslated region; QRE, quaking response element; QKI, quaking protein.

Major psychiatric disorders, represented by schizophrenia, have been well-recognized as highly polygenic based on genetic epidemiological findings at the population level (Gottesman and Shields, 1967). A recent large-scale GWAS identified more than 100 genetic loci in Schizophrenia Working Group of the Psychiatric Genomics Consortium (2014) and Li et al. (2017), which contained 332 Reference Sequence annotated genes (Birnbaum et al., 2015). When cross-referenced to microarray probes in the BrainCloud data set, 239 genes were further mapped out (Birnbaum et al., 2015), which included both protein coding genes and non-coding genes. Notably, most of these genes were not cell-type-specific (Mckenzie et al., 2018). Except for the GWAS data, recent whole exome sequencing studies have further identified some rare *de novo* single nucleotide and insertion/deletion variants in schizophrenia (Fromer et al., 2014; Purcell et al., 2014). If the genetic alterations occur in coding sequence, they may cause a destabilization of the protein conformation and aberrant posttranslational modifications (Figure 1; Ishizuka et al., 2017), thereafter resulting in functional deficits within various cell types.

In recent years, substantial functional studies of risk genes focused on neuronal function (Owen et al., 2016). Even though previous clinical studies demonstrated that several oligodendroglia and myelin related genes were significantly associated with major mental illnesses, such as MAG (Wan et al., 2005; Voineskos et al., 2013), Olig2 (Georgieva et al., 2006; Mitkus et al., 2008), and CNP (Voineskos et al., 2008), the function of a majority of schizophrenia related genes was poorly explored in oligodendroglia, which in turn hampers the understanding of pathological mechanisms of causal genes in oligodendroglia.

## Non-Coding Variants Affect Chromatin Accessibility and Gene Transcription

Non-synonymous protein coding changes cannot explain the majority of disease related genetic variants. Recent study found that the majority of GWAS hits occur within intergenic and intronic regions of the genome (Hindorff et al., 2009). Moreover, among these non-coding variants, 85% of non-synonymous variants and more than 90% of STOP gain and splice-disrupting variants are in low frequency (below 0.5%), and could be highly deleterious (Abecasis et al., 2012). For example, alterations to splicing site sequences, nucleotides adjacent to splice junctions and splicing regulatory sequences can severely influence gene splicing (Reble et al., 2018), which may explain global changes in alternatively spliced transcripts of risk genes (e.g., ERBB4 and DISC1) in psychiatric disorders.(Figure 1; Nakata et al., 2009; Chung et al., 2018).

In fact, some genetic effects owe to not only the common single nucleotide polymorphisms in GWAS, but also rare variants (Yu et al., 2018), copy-number variations (Malhotra and Sebat, 2012) and other types of mutations (Hall et al., 2015). A previous study identified schizophrenia related rare variants concentrated in regions of promoters and enhancers, but not insulators (Duan et al., 2014). Another study further found individuals harboring rare variants in conserved transcription factor binding motifs, untranslated regions of genes and non-coding RNAs (Abecasis et al., 2012). Therefore, these risk alleles could result in differential transcription factor binding and chromatin accessibility, implying their subsequent effects on the regulation of gene expression (Zhang et al., 2018). Moreover, recent DNaseI sequencing was applied to analyze chromatin accessibility at a genome-wide level, which further discovered genetic variants that modify chromatin accessibility which in turn may disrupt *cis* regulatory elements. These are major mechanisms that could explain how genetic alteration led to gene expression differences (Degner et al., 2012; Maurano et al., 2012).

Open chromatin, which is accessible for DNA-binding proteins, plays key roles in securing ordered spatiotemporal regulation of gene expression. Notably, some studies found that most of the identified open chromatin regions were enriched in promoters, enhancers and well-defined cell-type markers. Moreover, these regions were differentially accessible between neurons and non-neuronal cells, which may be related to the fact that neuronal open chromatin regions were more evolutionarily conserved and were enriched in distal regulatory elements as compared with non-neuronal cells (Fullard et al., 2017). Even though the precise molecular mechanism underlying this cell-type-specific discrepancy is not clear at present, it raises the possibility that genetic variants could exhibit cell-type-specific effects and differentially affect neuronal function and oligodendroglia function. Besides, the chromatin accessibility of oligodendroglia differentiation inhibitors, dynamic expression of transcription factors and non-coding RNAs were totally opposite or different between oligodendroglia progenitor cells and mature oligodendroglia (Emery and Lu, 2015), which shed light on the possibility that genetic variants occurring in these factors could differently interfere with oligodendroglia function at various developmental stages.

## Epigenetic Dysregulation of Risk Genes Affects Oligodendroglia Development and Myelination

Oligodendroglia differentiation and myelination are tightly controlled by epigenetic regulation. The transition from progenitor cells to mature oligodendroglia is characterized by a rapid and substantial chromatin remodeling (Nielsen et al., 2002; Liu et al., 2012), which is governed by epigenetic regulators included in the histone modifications and DNA methylation. Besides, the transcriptional activators and repressors negotiate through the underlying chromatin organization and play critical roles during the specification, differentiation and myelination of oligodendroglia (Emery and Lu, 2015). These findings suggest that dysregulation of the epigenetic regulators and transcription factors may impair oligodendroglia development and myelination.

In fact, some oligodendroglia specific risk factors (e.g., OLIG2, SOX10, and CNP), which are crucial for oligodendroglia development and myelination, are robustly dysregulated in major mental illnesses (Tkachev et al., 2003; Kato and Iwamoto, 2014). Recent genome-wide methylation analysis revealed overall disease related differential methylation of 817 genes in promotor regions (Wockner et al., 2014), which also included some oligodendroglia specific risk genes. These findings imply aberrant epigenetic mechanisms that regulate the expression of key risk genes in oligodendroglia. In particular, the DNA methylation of SOX10 was associated with oligodendroglia dysfunction in schizophrenia (Iwamoto et al., 2005), suggesting that epigenetic mechanisms causing functional deficit of risk genes could increase vulnerability of oligodendrocyte dysfunction during the pathogenesis.

Furthermore, several studies found a dysregulation of epigenetic regulators in the brains of psychiatric cohorts, including histone modification factors (e.g., histone acetyltransferase, histone deacetylase), DNA methylation factors (e.g., DNA methyltransferase, DNA methylase, and DNA demethylase) and microRNAs (Nestler et al., 2016). Therefore, if the epigenetic regulators were dysregulated in oligodendroglia, they may lead to, at least in part, the abnormal expression of oligodendroglia specific risk genes. However, as the epigenetic regulators are commonly expressed within various cell types, whether the aberrant epigenetic mechanisms somehow exhibit cell-type-specificity is vastly unclear.

In our previous study, we found that histone acetylation could affect transcription of FEZ1, a well-defined schizophrenia risk gene (Yamada et al., 2004; Kang et al., 2011), in oligodendroglia rather than in neurons (Chen et al., 2017), which might be due to variable contribution of transcription factor binding, providing an example of oligodendroglia-specific epigenetic regulation of risk genes. However, much of the epigenetic landscape remains unexplored in both neurons and oligodendroglia. Therefore, in order to deeply analyze the abnormal epigenetic modifications in nuclei and further understand the cell-type-specificity issue, chromatin modification and transcriptional profiling analyses could be applied to neuronal and oligodendroglia nuclei, which can be technically isolated from frozen postmortem human brain by fluorescence activated nuclear sorting (FANS) (Fullard et al., 2017).

## Gene Transcription is a Major Aspect Affected in Oligodendroglia, Likely Cooperating With Epigenetic Dysregulation

Strikingly, in a recent study, researchers analyzed genetic variants in glial type-specific schizophrenia risk genes by cross-referring GWAS data to previously published microarray database, and identified 1650 schizophrenia associated genes highly enriched in oligodendroglia (Goudriaan et al., 2014). Furthermore, the functional gene set analysis revealed three oligodendroglia-specific gene sets that were significantly associated with schizophrenia, including lipid metabolism, oxidation-reduction, and gene transcription (Goudriaan et al., 2014). Notably, the gene transcription set was the largest one and account for 47% of all disease related genes in oligodendroglia. Besides, the association between oligodendroglia gene transcription and disease depended on accumulated effects of multiple genes rather than the effects of a few genes (Goudriaan et al., 2014), implying that the transcription regulators in oligodendroglia could coordinate to play functional roles during the pathogenesis.

We further analyzed aforementioned gene transcription set, which included 123 genes in total. As described in Entrez Gene database^1^, these genes were involved in the histone modifications, transcription coactivation/corepression, transcription initiation and chromatin remodeling. Moreover, 59% of these genes were well-defined transcription factors. As the transcription factors are crucial for oligodendroglia differentiation and myelination (Emery and Lu, 2015), both the genetic deficit and epigenetic dysregulation of them may thereafter cause some functional deficits of risk genes and ultimately lead to the abnormalities of oligodendroglia and/or white matter.

## Molecular Network of Risk Genes in Oligodendroglia is Connected by Epigenetically Regulated Transcription and Post-Transcriptional Mechanisms

However, whether and how the oligodendroglia specific risk factors, including the above-mentioned transcription factors, coordinate to affect oligodendroglia function and myelination, except for their individual roles, is vastly unclear. For example, HDAC activity is necessary for oligodendroglia differentiation (Conway et al., 2012). When overall histone acetylation was changed in oligodendroglia, the expression of multiple psychiatric disorders related factors was altered (Conway et al., 2012; Chen et al., 2017). In our previous study, by inhibiting histone deacetylation in oligodendroglia, we demonstrated a sophisticated molecular orchestra in oligodendroglia regulating the expression of risk gene FEZ1 (Chen et al., 2017). Firstly, HDAC inhibition directly increased histone acetylation at the Fez1 promoter, therefore opened the chromatin at this DNA region. Secondly, HDAC inhibition in oligodendroglia altered the expression of some psychiatric diseases related transcription activators and repressors, which may cause imbalance of transcription activity and thereafter indirectly regulate FEZ1 transcription. Thirdly, the transcriptional repressor exhibited inhibitory roles on activators, which indeed fastened the cross-talk network of various transcription factors. Together with another study (Deng et al., 2017), these findings present an coordination between HDAC mediated chromatin remodeling and transcription factors, raising an intriguing possibility that DNA accessibility of risk genes and oligodendroglia-specific transcription factor orchestra may functionally interplay to regulate oligodendroglia function during the pathogenesis.

Besides epigenetic factors involved in gene transcription, some post-transcriptional regulators are also well-recognized as risk factors in oligodendroglia. For example, substantial evidence supports that quaking is a glia-expressed schizophrenia risk factor (Aberg et al., 2006a,b). The quaking protein, which controlled nuclear export, stability and translation of their bound mRNAs in oligodendroglia (Zhao et al., 2006; Bockbrader and Feng, 2008), was essential for oligodendroglia and myelin development (Chen et al., 2007). Except for the well-known myelin-related mRNAs involved in schizophrenia (Aberg et al., 2006a), we found that quaking protein can regulate FEZ1 mRNA stability through directly binding to FEZ1 mRNA at 3′UTR in the cytoplasm of oligodendroglia (Chen et al., 2017), hence connecting the function of two risk genes in oligodendroglia at the level of post-transcriptional regulation. Taken together, these studies provide an example how multiple risk factors in oligodendroglia are functionally connected in schizophrenia at transcriptional and post-transcriptional level (Figure 1), abnormalities of which may underlie oligodendroglia susceptibility and white matter etiology.

## Conclusion

Genetic alterations, as a large component of mental disease etiology, are not only affecting neuronal function, but also myelinating oligodendroglia. Moreover, these alterations need to induce functional effects through interaction with epigenetic regulators. Genetic variants located in gene coding regions could lead to aberrant protein conformation and posttranslational modifications. Genetic variants within non-coding regions are mostly included in cis regulatory elements including promoters and enhancers, and show deleterious effects by affecting chromatin accessibility and transcription factor binding motif (Figure 1).

Transcription and epigenetic regulation are important for oligodendroglia development, and are also affected during oligodendroglia pathogenesis. Ordered chromatin remodeling and transcription factor expression pattern during oligodendroglia development and myelin formation raise the possibility that genetic alterations and epigenetic dysregulations may exhibit functional effects at specific developmental stage based on the expression pattern and functional needs of the epigenetic factors. Moreover, multiple risk factors in oligodendroglia could functionally interplay at transcriptional and post-transcriptional level to affect oligodendroglia function during the pathogenesis, suggesting that malfunction of a molecular orchestra involving distinct risk factors may lead to the common pathophysiology of psychiatric diseases.

## Author Contributions

XC and JG had designed this study and drafted the manuscript. All authors contributed to reviewing and editing the final manuscript.

## Conflict of Interest Statement

The authors declare that the research was conducted in the absence of any commercial or financial relationships that could be construed as a potential conflict of interest.

## Footnotes

1 http://www.ncbi.nlm.nih.gov/gene
